# Repellent and insecticidal efficacy of a new combination of fipronil and permethrin against stable flies (*Stomoxys calcitrans*)

**DOI:** 10.1186/s13071-015-0688-6

**Published:** 2015-01-26

**Authors:** Becky Fankhauser, Jennifer P Irwin, Morgan L Stone, S Theodore Chester, Mark D Soll

**Affiliations:** Merial Limited, 3239 Satellite Blvd, Duluth, GA 30096 USA

**Keywords:** *Stomoxys calcitrans*, Stable flies, Dogs, Permethrin, Fipronil, Repellency, Insecticidal efficacy

## Abstract

**Background:**

A laboratory study was conducted to assess the repellent and insecticidal efficacy of a new combination of fipronil and permethrin (Frontline Tri- Act®/Frontect® Merial) against *Stomoxys calcitrans* (stable flies)*.*

**Methods:**

Sixteen dogs were allocated to two treatment groups. Eight dogs were treated with a new topical spot-on formulation containing 6.76% w/v fipronil + 50.48% w/v permethrin on Day 0 and eight dogs served as untreated controls. Each dog was exposed to approximately 100 stable flies on Days 1, 7, 14, 21, 28 and 35. After a one-hour exposure period, live flies were carefully aspirated into a vial, anesthetized with CO_2_ and crushed to determine feeding status (fed or unfed). Any dead flies remaining on the dog or in the cage were crushed to determine feeding status and counted as fed or unfed. Repellency was defined as the percent reduction in the number of fed flies in the treated group as compared to the untreated control group, and insecticidal efficacy was defined as the reduction in the number of live flies at the end of each exposure period in the treated group as compared to the control group.

**Results:**

Percent repellency was ≥96.6% through Day 28, and 88.7% on Day 35. Percent insecticidal efficacy was ≥ 98.3% through Day 35.

**Conclusions:**

A single topical administration of a new combination of fipronil and permethrin provides protection (repellency and insecticidal efficacy) from *S. calcitrans* on dogs for at least 5 weeks.

## ABRÉGÉ

**Contexte:**

Une étude expérimentale a été conduite afin de mesurer l’efficacité répulsive et insecticide d’une nouvelle combinaison de fipronil et de perméthrine (Frontline Tri- Act®/Frontect®, Merial) contre *Stomoxys calcitrans* (mouche piqueuse).

**Méthode:**

Seize chiens ont été répartis en deux groupes. Huit chiens ont été traités avec une nouvelle formulation topique en spot-on contenant du fipronil à 6,76 % w/v + perméthrine à 50,48 % w/v au jour 0. Huit chiens servaient de témoins non traités. Chaque chien a été exposé à environ 100 mouches piqueuses aux jours 1, 7, 14, 21, 28 et 35. Après une période d’exposition d’une heure, les mouches vivantes ont été aspirées délicatement dans un récipient, anesthésiées au CO_2_ et écrasées afin de déterminer leur statut de gorgement (gorgées ou non). Toutes les mouches mortes restant sur le chien ou dans la cage ont été aussi écrasées pour déterminer leur statut de gorgement, et comptabilisées comme gorgées ou non. L’activité répulsive a été définie comme le pourcentage de réduction du nombre de mouches gorgées dans le groupe traité comparé au groupe témoin non traité, et l’efficacité insecticide est définie comme la réduction du nombre de mouches vivantes à la fin de chaque période d’exposition, dans le groupe traité comparé au groupe témoin non traité.

**Résultats:**

L’activité répulsive a été ≥ 96,6 % jusqu’au jour 28, et 88,7 % au jour 35. Le pourcentage d’efficacité insecticide a été ≥ 98,3 % jusqu’au jour 35.

**Conclusion:**

Une administration topique unique de la nouvelle combinaison de fipronil et de perméthrine offre une protection des chiens (efficacité répulsive et insecticide) contre *S. calcitrans* pour au moins 5 semaines.

## Background

The control of ectoparasites on pets is an important concern for owners. While the control of fleas, ticks, sandflies and mosquitoes receives frequent attention, the control of other biting flies is also of importance for pets. In particular, the stable fly (*Stomoxys calcitrans)* and the fruit fly (*Phortica variegata*), a vector of *Thelazia callipaeda* in domestic and wild carnivores (*i.e.*, dogs and cats) as well as humans, have an important veterinary impact [1,2].

*Stomoxys calcitrans*, or the stable fly, is of particular importance to cattle and horses, but also to dogs and humans. Stable flies are the most common blood-feeding fly around farm animals and the females lay their eggs in moist, decaying organic material, such as piles of lawn clippings, damp hay, grain or animal manure [1,3]. Both male and female stable flies feed on blood once or twice a day, depending on the ambient temperature [1]. Stable flies attack and bite dogs, particularly dogs that live near and around horses or cattle. They can be a serious concern for dogs and are even known as the “dog fly” in some geographic areas [4]. Stable flies most frequently bite the ears, muzzle and nose, and are the most common cause of fly bite dermatitis in dogs. The bites can be painful and can cause significant irritation and restlessness as well as small ulcers covered by hemorrhagic crusts [4-6]. In severe cases, the ears may have significant bleeding, become infected, become necrotic from the irritation, and may eventually become permanently disfigured [4,6]. Penned hunting dogs are especially vulnerable to stable flies due to their inability to escape the flies’ persistent attacks [4]. Stable flies can also be a problem in kennels and in one study of 10 greyhound kennels in Kansas, USA, an average of 177 stable flies per day were caught in each kennel and up to 50% of dogs in these kennels had open or crusted ulcers indicative of stable fly feeding [7]. Stable flies also readily attack people, typically on the lower part of the legs [6]. Pyrethroids and other repellents, as well as efforts to control fly breeding sites, are used to control stable flies in affected areas [1].

Permethrin belongs to the Type 1 class of pyrethroids, which are insecticides and acaricides with repellent activity. Permethrin has been used for years for the control of ectoparasites on both companion and production animals as well as humans. Both single-active formulations as well as combination products have been shown to effectively control stable flies [6,8-10].

Fipronil belongs to the phenylpyrazole family and has both insecticidal and acaricidal activity. Fipronil has been used for years to control fleas and ticks on companion animals [11-14].

A novel combination of 6.76% w/v fipronil and 50.48% w/v permethrin (Frontline Tri- Act®/Frontect®, Merial) has been developed for use as a monthly topical solution for dogs to provide broad spectrum ectoparasite control. This study was conducted to assess the repellent and insecticidal efficacy of this combination against *S. calcitrans.*

## Methods

The study was conducted in accordance with Good Clinical Practices (GCP) as described in *International Cooperation on Harmonisation* of Technical Requirements for Registration of *Veterinary* Medicinal Products (VICH) guideline GL9 [15].

### Animals

Eighteen dogs were exposed to stable flies prior to enrollment in the study to assess their susceptibility to fly feeding, and a veterinary examination was performed to ensure that all dogs were healthy and suitable for inclusion in the study. The 16 dogs deemed healthy and suitable for the trial and with the highest fed fly count were used in the study. The 16 dogs were ranked by descending fed fly count within sex and eight blocks of two dogs each were formed. Within blocks, each dog was randomly allocated to one of two treatment groups.

Eight male and eight female healthy Beagle dogs (13.3 to 14.6 months of age) were used in this study. The dogs had not been exposed to ectoparasiticides in the 3 months prior to the study and were managed with due regard for their well-being, in accordance with Merial Institutional Animal Care and Use Committee requirements. Dogs were housed individually in indoor cages with controlled environmental conditions. The dogs were observed daily for any health changes throughout the study.

### Treatment

Dogs in Group 1 received no treatment and served as untreated controls. Dogs in Group 2 were treated once with a topical formulation containing 6.76% w/v fipronil and 50.48% w/v permethrin. Each dog was dosed with the minimum dose of the product (0.1 mL/kg: 6.76 mg/kg fipronil and 50.48 mg/kg permethrin) once topically. The total volume of the product was divided into two approximately equal fractions and placed on the skin on the midline of the neck. One fraction was applied between the base of the skull and the shoulder blades and the other was applied at the front of the shoulder blades. All dogs were observed hourly for any adverse reaction for 4 hours post-treatment.

### Stable flies

Laboratory-reared *S. calcitrans* were used in this study. The flies were obtained from New Mexico State University, USA, from a colony that originated in Florida, USA. The flies arrived predominantly as pupae 6 to 7 days prior to each exposure. Emerging flies were fed a 10% sucrose solution and whole citrated bovine blood. Two days prior to dog exposure, the blood was withheld and flies were allowed to feed on sucrose solution only. The sucrose solution was removed and flies were fasted the night before the exposures. The adult flies were approximately 3 to 10 days old when exposed to the dogs.

### Stable fly exposures and counts

Each dog was exposed to approximately 100 (average = 101.6, range = 86–118) *S. calcitrans* on Days −8, 1, 7, 14, 21, 28 and 35. Prior to exposure, each dog was anesthetized with a combination of butorphanol (0.2 mg/kg; Torbugesic®, Zoetis), dexmedetomidine (0.02 mg/kg; Dexdomitor®, Orion Pharma) and midazolam (0.2 mg/kg;Hospira, Inc. and Sagent Pharmaceuticals) administered intramuscularly. Additional amounts of the anesthetics were administered as needed to maintain anesthesia for the duration of the exposure period. Once anesthetized, each dog was placed into an individual fly proof exposure cage and approximately 100 *S. calcitrans* were released into the exposure cage. After the one hour exposure period, live flies were carefully aspirated into a vial (one per dog). The dog was removed from the cage, checked for any flies remaining on the fur and given a reversal agent (atipamezole, 0.1 to 0.2 mg/kg, administered intramuscularly; Antisedan®, Orion Pharma). Any dead flies remaining on the dog or in the cage were crushed to determine feeding status and counted as fed or unfed. The live flies in each vial were anesthetized with CO_2_ and crushed to determine feeding status (fed or unfed).

All personnel conducting fly counts and health observations were blinded to treatment groups. After treatment, different personnel handled treated and untreated dogs and separate equipment (aspirators, exposure cages) was used for each treatment group to prevent cross-contamination between dogs. If personnel handled dogs from different groups, disposable lab coats and gloves were worn and changed between groups. The exposure cages were thoroughly cleaned between each exposure.

### Data analysis

#### Percent fly repellency

Percent repellency was defined as the percent reduction in the number of fed flies in the treated group as compared to the untreated control group. The total numbers of fed (live + dead) flies at the end of each exposure period were transformed to the natural logarithm of (count + 1) for calculation of geometric means (GM) by treatment group. Parasite counts were transformed to the logarithm scale prior to analysis to stabilize the animal-to-animal variability and because the histogram of the transformed counts tends to be more symmetric than the histogram of the untransformed counts. Parasite counts were transformed to the logarithm scale prior to analysis to stabilize the animal-to-animal variability and because the histogram of the transformed counts tends to be more symmetric than the histogram of the untransformed counts. Percent repellency at each post-treatment exposure day was calculated as 100 x [(C-T)/C], where C is the GM of the control group and T is the GM of the treated group.

#### Percent insecticidal efficacy

Percent insecticidal efficacy was defined as the reduction in the number of live flies at the end of each exposure period in the treated group as compared to the control group. The number of live flies after each exposure was transformed to the natural logarithm of (count + 1) for calculation of GM by treatment group as previously described [16]. Parasite counts were transformed to the logarithm scale prior to analysis to stabilize the animal-to-animal variability and because the histogram of the transformed counts tends to be more symmetric than the histogram of the untransformed counts. The 1 is added to the counts to handle counts that are zero. Percent insecticidal efficacy of the treated group compared to the untreated control group at each time point was calculated as 100x[(C-T)/C], where C is the GM of the control group and T is the GM of the treated group.

For both repellency and insecticidal efficacy, the treated group was compared to the untreated control group using the Friedman rank test with blocks defined as the allocation blocks [17]. The testing was two-sided and used a significance level of 5%. The analyses were performed using SAS® Version 9.1.3.

## Results

No adverse reactions to the treatment were observed in any dog during the study, including the 4-hour period after treatment. Four dogs in the untreated control group developed suspected allergic reactions (hives, facial swelling) following one or more of the *S. calcitrans* exposures. The affected dogs recovered after treatment with diphenhydramine (Benadryl®, West-Ward Pharmaceuticals). One dog in the untreated control group and two dogs in the treated group developed hypersalivation following administration of the anesthesia reversal agent, which resolved without treatment.

The stable fly challenge was robust on all exposure days as evaluated by the number of live flies and the number of fed flies in the untreated control dogs at the end of each exposure period. The GM of live flies in the untreated control group ranged from 92.9 to 101.3 and the number of fed flies ranged from 75.1 to 91.6 during the study (Tables 1 and 2).

**Table 1:** **Percent repellency of** ***Stomoxys calcitrans*** **in dogs treated with a new combination of fipronil and permethrin**

	**Number of fed stable flies (GM)**		
**Exposure day**	**Untreated control dogs (n = 8)**	**Treated dogs (n = 8)**	**Repellency (%)**
1	83.1	0.0	100*
7	83.5	0.0	100*
14	75.1	0.6	99.2*
21	79.1	2.2	97.3*
28	85.9	2.9	96.6*
35	91.6	10.4	88.7*

*Significant difference between the population means of the treated and control groups (*p* = 0.005).

GM: geometrical mean of the natural logarithm of (count + 1).

**Table 2:** **Percent insecticidal efficacy of** ***Stomoxys calcitrans*** **in dogs treated with a new combination of fipronil and permethrin**

	**Number of live stable flies (GM)**		
**Exposure day**	**Untreated control dogs (n = 8)**	**Treated dogs (n = 8)**	**Insecticidal Efficacy (%)**
1	92.9	0.0	100*
7	95.5	0.1	99.9*
14	95.8	0.6	99.4*
21	96.2	0.4	99.6*
28	101.3	1.8	98.3*
35	98.6	1.6	98.3*

*Significant difference between the population means of the treated and control groups (*p* = 0.005).

GM: geometrical mean of the natural logarithm of (count + 1).

### Fly repellency

The GM number of fed flies in each group and the percent repellency on each exposure day is shown in Table 1. Repellency was 100% on Days 1 and 7, 99.2%, 97.3% and 96.6% on Days 14, 21 and 28, respectively, and 88.7% on Day 35. There was a significant difference between the population means of the treated and control groups at every time point (*p* = 0.005).

### Fly insecticidal efficacy

The GM number of live flies in each group and the percent insecticidal efficacy on each exposure day is shown in Table 2. The percent insecticidal efficacy was 100% on Day 1 and 99.9%, 99.4%, 99.6%, 98.3% and 98.3% on Days 7, 14, 21, 28 and 35, respectively. There was a significant difference between the population means of the treated and control groups at every time point (*p* = 0.005).

## Discussion

The results of this study demonstrate that a single topical treatment of the new combination of fipronil and permethrin (Frontline Tri-Act®/Frontect®) provides excellent repellency and insecticidal efficacy for at least 5 weeks. The product provided immediate effects after administration as demonstrated by 100% repellency and insecticidal efficacy one day after treatment. Both effects persisted for over 1 month with 96.6% repellency 28 days after treatment and 88.7% repellency 35 days after treatment. Insecticidal efficacy remained over 98% for 35 days after treatment.

The repellent effects of the combination of fipronil and permethrin will help prevent painful bites as well as more severe sequelae such as bleeding, infection and necrosis for at least 5 weeks after treatment. In addition to the direct benefits from the repellent effect, the insecticidal effects of the product may also help reduce overall stable fly populations in kennels or other situations where the flies rely on dogs as their sole source of blood. This reduction in fly population may further reduce the challenge to dogs in these areas.

The stable flies in this study provided a strong challenge to the product on each exposure day. A high percentage of the flies fed on the untreated control dogs on each day of the study, with 75.1 to 91.6 of approximately 100 flies having fed by the end of the exposure period. The viability of the flies was high with 92.9 to 101.3 of the flies remaining alive at the end of the exposure period. The percentage of flies that fed and survived the exposure period in this study was higher than previously reported by Fourie *et al.* [6]*,* in which an average of 12.6 of 50 flies took a blood meal during a 30-minute exposure period and 22.5 of 50 flies survived the exposure period. The reason for the higher feeding and survival rate in the current study is unknown but may be related to the source, age, rearing or holding conditions of the stable flies, longer exposure time or other reasons. Flies used in this study were younger and fasted from blood for a shorter time prior to exposure, which may explain some of the difference. Regardless of the reason for the high feeding rate in the current study, it demonstrates that the challenge was robust and a strong test of the product.

The repellency and insecticidal efficacy of the combination of fipronil and permethrin was higher than previously reported for a commercial permethrin-containing product. Fourie *et al.* [6] reported that repellency for an imidacloprid (10%) and permethrin (50%) combination was highest (90.2%) one day after treatment and least (82.1%) 4 weeks after treatment. Insecticidal efficacy in this same study varied between 77.2% and 78.7% during the first 8 days after treatment and then decreased to 39.2% by the end of the study (29 days after treatment). The reason for the difference in repellency and insecticidal efficacy of the combination of fipronil and permethrin may be explained by the fact that fipronil has also been shown to have some effects on stable flies. It is possible that combining two active ingredients that both have activity against stable flies leads to improved inhibition of fly feeding for a longer duration.

## Conclusions

A single topical administration of a combination of fipronil and permethrin provided repellent (anti-feeding) protection from *S. calcitrans* on dogs for 5 weeks. The product also provided a high insecticidal efficacy against flies exposed to treated dogs for 5 weeks following treatment. The product has been shown to be safe for dogs and will provide reliable protection against a variety of ectoparasites, including stable flies, for at least 1 month.
